# Metagenomics-based analysis of microbial community structure and functional differences in fermented grains of Jiang-flavored baijiu from different production regions and policy recommendations for industrial development

**DOI:** 10.3389/fmicb.2025.1619035

**Published:** 2025-07-16

**Authors:** Jiaqi Shen, Yuanyan Hu, Yunjin Zhang, Lixia Li, Xiaofeng Deng, Manjing Chen, Laoda Li, Peiyun Xie, Mingbo Shao

**Affiliations:** ^1^Institute of Upland Food Crops, Guizhou Academy of Agricultural Sciences, Guiyang, China; ^2^School of Public Administration, Guizhou University of Finance and Economics, Guiyang, China; ^3^Guizhou Light Industry Technical College, Guiyang, China; ^4^Guizhou Academy of Social Sciences, Guiyang, China; ^5^Guiyang Healthcare Vocational University, Guiyang, China; ^6^Guizhou Bijie Distillery Co., Ltd., Bijie, China

**Keywords:** Jiang-flavored baijiu, fermented grains, metagenomics, microbial community structure, functional genes

## Abstract

**Introduction:**

Recently, some regions that originally focused on strong-flavor baijiu production started producing Jiang-flavored baijiu, providing a new perspective for studying the dynamic changes in the microbial community during brewing.

**Methods:**

This study used second-round fermented grains of Jiang-flavored baijiu from three Guizhou production regions (Renhuai, Duyun, and Bijie). By applying metagenomics technology and various analytical and statistical methods, we analyzed the community structures of bacteria and fungi in fermented grains, their functional genes, and their correlations with environmental factors.

**Results:**

We identified 1063 bacterial genera and 411 fungal genera. Although the dominant microbial species were similar across regions, their relative abundances differed significantly. *α*-diversity analysis showed that grains from the Bijie region had higher species richness and evenness indices, indicating the significant impact of geographical location and the strong-flavor baijiu-brewing background on microbial structure and composition. Analysis of similarity and the Wilcoxon rank-sum test revealed significant differences in the microbial communities of different regions, and we identified genera with large differences in abundance, such as *Desmospora* and *Kroppenstedtia* among bacteria, and *Pyrenophora* and *Blyttiomyces* among fungi. Based on our Kyoto Encyclopedia of Genes and Genomes (KEGG) database analysis, the Duyun region had a significantly higher abundance of metabolism-related genes at the tertiary KEGG level. Redundancy analysis showed that six environmental factors (relative humidity, daily temperature difference, elevation, annual mean temperature, extreme cold temperature, and annual precipitation) exerted complex effects on microbial functional genes in fermented grains. Carbon metabolism, antibiotic biosynthesis, and elevation were positively correlated with microbial functional genes. Actinobacteria are crucial for carbon metabolism, followed by Proteobacteria and Chloroflexi.

**Discussion:**

This study elucidated the structural and functional characteristics of microbial communities in second-round fermented grains of Jiang-flavored baijiu under production area transitions and proposed policy recommendations to promote the differentiated development of the baijiu industry.

## 1 Introduction

Baijiu, a traditional Chinese distilled spirit, holds a significant position in the global distilled spirits domain. It ranks among the world’s top six distilled spirits, along with whisky, vodka, brandy, rum, and gin (Sun, 2021; Qiao et al., 2023). Among various baijiu types, Jiang-flavored baijiu is notable for its distinct soy-sauce-like aroma, elegance, delicacy, and long-lasting aftertaste (Wang L. et al., 2021; Tu et al., 2022). Moreover, it provides a favorable post-consumption experience (Lin et al., 2023). Baijiu drinkers often report mild physical reactions, which contribute to their popularity. By 2023, the production volume of Jiang-flavored baijiu is projected to exceed 750,000 tons, accounting for 11.9% of the total national baijiu production. Notably, its profit accounted for 30.4% of the total profit of the baijiu industry (Quantu Jiangjiu Studio, 2024). Jiang-flavored baijiu, with a relatively small production proportion, generates nearly one-third of the industry’s total profit, highlighting its high value-added advantage. In recent years, the market for Jiang-flavored baijiu has been booming as consumer demand for high-quality baijiu has been increasing.

In addition to the traditional core production areas of Moutai Town and Xishui Town in Guizhou Province, the Jiang-flavored baijiu industry is flourishing in other regions of Guizhou. Driven by the prosperous development of the Jiang-flavored baijiu market, some well-known enterprises with a strong presence in the strong-flavored baijiu-brewing field have launched new production lines for Jiang-flavored baijiu. These enterprises aim to integrate into the rapidly expanding market segment of Jiang-flavored baijiu to capture market share and achieve economic benefits. However, the baijiu market continues to evolve. Specifically, the preferences of young consumers have become more personalized, and their demand for diverse baijiu flavors has increased. Traditional baijiu tastes may no longer be the sole popular option, as many consumers are now seeking baijiu products with varied tastes and flavors. This shift in taste reflects consumers’ higher expectations of baijiu flavors, brewing techniques, production environments, and taste experiences. Therefore, differentiated competition is critical to enhance product competitiveness.

The unique flavor profile of baijiu demonstrates its remarkable geographical dependence (Yuan et al., 2023). Despite similar raw material formulations and brewing techniques across different production regions, flavor differences remain notable, especially for Jiang-flavored baijiu, whose final products show distinct differences (Gong et al., 2023; Pan et al., 2023). The underlying reason for these distinct flavor variations can be primarily attributed to the distinctive fermentation process inherent in Jiang-flavored baijiu (Wang et al., 2016; Dai et al., 2020; Wang, 2022).

The brewing procedure for Jiang-flavored baijiu involves seven rounds of intricate fermentation, each consisting of two key stages: stacking and intercellular fermentation (Chen et al., 2024). In the stacking fermentation stage, the fermented grains are proportionally mixed with a large quantity of Daqu and water. Subsequently, the samples were placed in a semi-open drying space. They are allowed to ferment in a natural environment for a period ranging from 3 to 7 days (Wang H. et al., 2021). During this process, fermented grains attract and accumulate a vast array of microorganisms from the surrounding brewing milieu. As a result, functional microbial flora experience rapid growth, and a substantial number of flavor precursor substances are generated. These substances serve as the basis for the subsequent in-cell fermentation. Therefore, the growth and metabolic activities of functional microorganisms during the stacking fermentation stage are closely intertwined with the surrounding environmental factors and exhibit a high degree of dependence on environmental conditions (Xu et al., 2022; Zou et al., 2024). This dependence on the environment is a crucial factor that contributes to the distinct flavor profiles of Jiang-flavored baijiu produced in different geographical locations.

Previous studies have investigated the microbial communities in fermented grains from different climatic regions. For instance, a group of researchers conducted a meticulous comparative analysis of fungal communities in fermented grains from Guizhou, China, which has a humid subtropical climate, and Beijing, China, which has a temperate monsoon climate. These findings indicate that the dominant functional fungal communities in the fermented grains of the two regions exhibit substantial differences in both population composition and colony abundance (Ding et al., 2024). Nevertheless, there remains a dearth of research regarding alterations in microbial community structure induced by changes in topography and altitude within the same climate type. This knowledge gap is particularly pronounced considering the lack of understanding of how these factors influence the fermentation process and the resulting flavor profiles of baijiu. Furthermore, for enterprises that have long been dedicated to the production of strong-flavor baijiu and have only recently ventured into the production of Jiang-flavor baijiu, there is a lack of systematic research reports on the characteristics and change patterns of functional microorganisms within new production systems. Understanding these aspects is crucial for optimizing the brewing process, ensuring product quality, and developing unique flavor profiles for emerging Jiang-flavored baijiu production lines. Such research could potentially uncover novel fermentation strategies and microbial interactions that could enhance the competitiveness of these new entrants in the Jiang-flavored baijiu market.

Renhuai, Duyun, and Bijie, which are located in the northwestern, southern, and southwestern parts of Guizhou, respectively, possess distinct geographical features. A significant difference in altitude is evident among them, and elevation, as a key determinant, directly impacts temperature and air pressure, which can markedly affect the growth and metabolic activities of microorganisms during the fermentation of Jiang-flavored baijiu. The topography of these regions varies greatly, including mountains, plains, and valleys, creating diverse microhabitats for microbial colonization. The effective sunshine hours and diurnal temperature differences also vary between these regions. Longer hours of sunshine can promote certain chemical reactions in fermented grains, whereas larger diurnal temperature differences may affect the balance of microbial communities. In addition, forest vegetation coverage and types differ, contributing to the formation of unique microclimates. These environmental factors, either independently or interactively, can profoundly impact the microbial composition and function of fermented grains of Jiang-flavored baijiu.

Guizhou Province is the core production area for Jiang-flavored baijiu owing to its unique geography and climate. Improvements in the efficiency of the liquor industry have effectively stimulated the demand for sorghum brewing. Starting in 2020, Guizhou Province prioritized the waxy sorghum planting industry as a key commercial opportunity, with the authorities in Guizhou establishing sorghum planting demonstration sites in addition to Renhuai (a prominent production region) and gradually forming suitable production areas in northern, southwestern, and southern Guizhou. Although the fermentation characteristics of waxy sorghum production in various regions have been reported, there is a lack of information regarding the physicochemical properties and microbial community composition on the surface of the same variety sourced from different locations. Therefore, this study aimed to analyze the physicochemical properties of sorghum and investigate the structure of the microbial communities and dominant functional genes in sorghum from the production regions in Renhuai, Jinsha, and Duyun using high-throughput sequencing. To explore the factors influencing surface microorganisms, we collected soil samples from different production areas and downloaded the relevant meteorological data. This study provides information that supports the improvement of the product stability and competitiveness of baijiu enterprises through differential development. Moreover, this study provides an important scientific basis for evaluating microbial resources for brewing.

## 2 Materials and methods

### 2.1 Sample collection

The second round of fermentation is of great significance in the complex brewing process of Jiang-flavored baijiu, which consists of eight rounds of fermentation. Second-round fermented grains are crucial intermediate products in the production chain. The fermentation quality at this stage not only affects the yield but also has a decisive influence on the flavor and quality of the subsequent round of baijiu. Although the overall baijiu-making process remains consistent across all rounds, the base baijiu obtained from second-round fermented grains has unique flavor characteristics. At this stage, the typical rich and mellow Jiang flavor has not yet developed, but the product already shows relatively light, spicy, and sour flavors, making it interesting to study the flavor formation mechanism in the early stages of Jiang-flavored baijiu production (Zou et al., 2024; Wang et al., 2025). A detailed comparison of the microbial community structures in second-round fermented grains from the three selected origins, along with the accurate identification of functional microbial groups and their potential genetic functions, can provide new insights into the microbial diversity and rich genetic resources across different production areas in Guizhou Province and lay a solid foundation for further in-depth research on how microbial communities respond to environmental changes and adapt to diverse ecological conditions during the fermentation process.

Fermented grain samples were systematically collected from three distinct production regions in the Guizhou Province in China: Renhuai, Bijie, and Duyun. These regions serve as the basis for leading enterprises in renowned baijiu production zones in Guizhou, which are highly esteemed within the industry. The sampling period spanned from April to June 2022. Given the significant climatic differences among these regions, the commencement time of initial raw material processing varied substantially. This variation in sand-laying time directly resulted in differences in the sampling time of the second-round fermented grains. All collected fermented grain samples were derived from the second round of fermentation, with a specific focus on the third day of the stacking fermentation stage. To ensure representativeness, fermented grain samples were randomly selected from the top, middle, and bottom layers of the fermented grain piles for each baijiu-producing enterprise. Five sampling points were designated for each layer, totaling 15 sampling points per enterprise. Subsequently, each set of samples from the different points was crushed and thoroughly mixed. The sampling procedure was replicated three times for each of the three enterprises, and nine enterprise-level samples were obtained, including 135 individual samples collected from different locations. Immediately after sampling, the fermented grain samples were transferred to aseptic packaging and transported to the laboratory using a cold chain system. This strict transportation protocol was implemented to preserve the integrity of the samples and prevent any potential contamination or degradation of the microbial communities within the fermented grains, which could affect the accuracy of subsequent analyses.

### 2.2 DNA extraction, amplification, and sequencing

A metagenomic library was constructed using a TruSeq DNA PCR-Free Library Preparation Kit (Illumina, San Diego, CA, USA). The metagenomic library quantity was evaluated using a fluorometer (Qubit V.2.0). Metagenomic sequencing was conducted at the Realbio Technology Center (Shanghai, China) using an Illumina HiSeq 4,000 platform with 100 bp paired-end reads. Host contamination was removed from the raw sequencing file using KneadData by aligning the genome. The remaining reads were trimmed to quality using Trimmomatic software (v0.33). Contigs of at least 300 bp were selected as the final assembly result.

### 2.3 Climate data

The climate data collected included average monthly temperature, effective accumulated temperature, diurnal temperature variation, precipitation, and relative humidity, which were obtained from the daily value dataset of surface climate data provided by the China Meteorological Science Data Sharing Service Network.

### 2.4 Data and statistical analysis

To study the functional composition of fermented grain microbial communities, metagenomic sequence reads that had undergone the removal of host genomes were assembled using MEGAHIT (version 1.0.6) (Li et al., 2015; Yang et al., 2019) with the default parameters. The assembly was predicted using QUAST (Gurevich et al., 2013). The DIAMOND program (v2.0.13) was used to map the amino acid sequences of the gene catalog onto the proteins in the Kyoto Encyclopedia of Genes and Genomes (KEGG) database (release 88.0). The KEGG database has been used to understand biological systems, resulting in an understanding of the functions of the microbiome in the host (Kanehisa, 2004). To analyze carbon metabolism, we first mapped the metagenomic KEGG results to carbon cycle-related functions and genes in a database curated by Biomarker Technologies. For each relevant function or gene in the database, we summed the abundances of the corresponding matched elements from the metagenomic KEGG results to obtain their overall abundances. Subsequently, we visualized these abundances by generating a heatmap that effectively presented the distribution and variation of carbon metabolism-related functional genes and pathways.

The *α*-diversity, including the Shannon index in each sample, was calculated to evaluate the corresponding diversities using R (v3.6.3). An analysis of similarities (ANOSIM) based on the Bray–Curtis distance was also performed. This analysis enabled an analysis of the significance of the differences in terms of species and genes. It also generated corrected *p*-values (false discovery rate, FDR). Redundancy analysis (RDA), an ordination technique that evolved from correspondence analysis, was developed using a linear model. To determine the most appropriate analytical model, a detrended correspondence analysis (DCA) was performed using species sample data. DCA is crucial because it provides insights into the nature of ecological gradients within the dataset. Specifically, the length of the gradient along the first axis of the DCA results is a key indicator of model selection. In our case, the value of the first-axis gradient length was less than 3.0. This finding suggests that the relationship between the variables can be adequately described using a linear model. Consequently, the RDA model was selected for subsequent in-depth analyses. All analyses and generation of corresponding graphical representations were performed using the vegan package within the R statistical environment (version 3.6.3). Random Forest, a prominent algorithm in machine learning, functions as a classifier composed of an ensemble of decision trees. This algorithm offers a robust approach to identifying the most influential species categories for classification tasks by leveraging the predefined classification of the original samples. By integrating the collective wisdom of multiple decision trees, Random Forest can efficiently and precisely identify key species that contribute significantly to the classification process. Analyses and graphing were performed using the Random Forest package in R (version 3.6.3).

## 3 Results and discussion

### 3.1 Bacterial and fungal community composition

**Figure 1:**
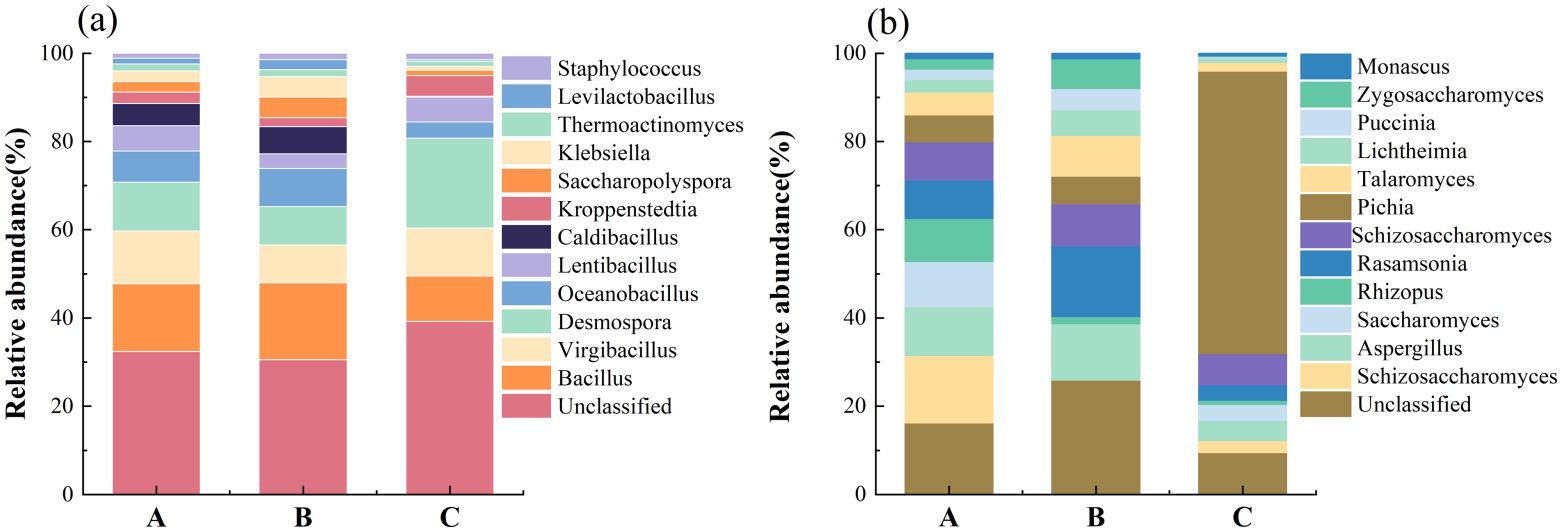
Community composition of **(a)** bacteria and **(b)** fungi at the genus level.

Following a rigorous and systematic quality control process of the sequencing data, the resulting clean read data are presented in Supplementary Table S1. In Renhuai, 41,037,595 clean reads were obtained; in Bijie, 40,323,969 clean reads were obtained; and in Duyun, 40,815,905 clean reads were generated. All samples were then comprehensively analyzed and identified. Through this in-depth examination, 1,063 bacterial genera and 411 fungal genera were identified. The structure of the dominant microorganisms (defined as those with a relative abundance >1%) in the fermented grains from different production regions is visually depicted in the form of bar charts in Figure 1. Overall, although the dominant bacterial and fungal species were generally consistent across production regions, notable differences were observed in their relative abundances.

In the Renhuai production region, a diverse array of bacteria was identified as the dominant members of the microbial community. *Bacillus* exhibited the highest average relative abundance among bacteria, reaching 12.1% (the relative abundance reporting convention remained consistent throughout the text). *Virgibacillus* had a relative abundance of 9.47%, followed by *Desmospora* at 8.72%, *Oceanobacillus* at 5.57%, and *Lentibacillus* at 4.58%. *Caldibacillus* accounted for 3.94%, *Kroppenstedtia* for 2.05%, *Saccharopolyspora* for 1.90%, *Klebsiella* at 1.89%, and *Thermoactinomyces* at 1.27%. *Schizosaccharomyces* was the most prominent fungal community, with a relative abundance of 14.1%, followed by *Aspergillus* at 13.4%, *Saccharomyces* at 9.64%, *Rhizopus* at 8.97%, and *Rasamsonia* at 8.54%.

In contrast, the microbial composition in the Bijie production region showed some differences. Among these bacteria, *Bacillus* was dominant, with a relative abundance of 12.5%. *Desmospora* had a relative abundance of 6.24%, *Oceanobacillus* at 6.19%, *Caldibacillus* at 4.42%, *Saccharopolyspora* at 3.38%, *Klebsiella* at 3.36%, and *Lentibacillus* at 2.43%. Among the fungi, *Schizosaccharomyces* was the most abundant, with a relative abundance of 21.7%, followed by *Paecilomyces* at 13.4%, *Saccharomyces* at 10.5%, *Pichia* at 8.03%, and *Talaromyces* at 5.32%.

Notably, the microbial profile in the Duyun production region deviated further from those in the other two regions. Among these bacteria, *Desmospora* was dominant, with a relative abundance of 16.7%. The relative abundance of *Virgibacillus* was 8.97%, that of *Bacillus* was 8.42%, *Lentibacillus* at 4.65%, *Kroppenstedtia* at 3.85%, *Oceanobacillus* at 3.02%, and *Staphylococcus* at 1.21%. In the fungal community, *Schizosaccharomyces* had a relative abundance of 8.89%, followed by *Pichia* (6.62%), *Saccharomyces* (4.27%), *Rhizopus* (3.44%), and *Paecilomyces* (3.28%).

Overall, although the dominant bacterial and fungal species were consistent across different production regions, there were pronounced differences in their relative abundances. This phenomenon is likely attributable to the shaping effects of various factors, such as the environment of the production region and brewing techniques, on the microbial community. Regarding the bacterial community, the relative abundances of *Bacillus* in the Renhuai, Bijie, and Duyun production regions were 12.1, 12.5, and 8.42%, respectively. As a common genus of spore-forming bacteria, *Bacillus* spp. are ubiquitous in natural environments. During baijiu brewing, *Bacillus* spp. participate in various metabolic activities, such as the decomposition of carbohydrates and proteins, thereby providing an essential material basis for fermentation (Li et al., 2023). The Bijie production region is adjacent to the newly added Jiang-flavored baijiu workshop, near the strong-flavored baijiu workshop. This special environment may be influenced by strong flavor-brewing microorganisms. Common spore-forming bacteria and other strains in strong-flavor baijiu brewing may enter the Jiang-flavor baijiu workshop in the Bijie production region through the air and certain equipment, thus altering the relative abundances of bacteria in the fermented grains of this region to a certain extent. This has led to differences in the relative abundances of some bacteria, such as *Bacillus*, between the Bijie-producing region and other regions. Moreover, the relative abundances of bacteria such as *Desmospora* (6.24%) and *Oceanobacillus* (6.19%) in the Bijie production region differed from those in the Renhuai and Duyun production regions. This may be related to the unique local geographical environment, raw material characteristics, and cross-influence of microorganisms from the strong-flavor baijiu workshop in these regions.

The fungal community composition also differed among the production regions. The relative abundance of *Schizosaccharomyces* in the Bijie production region reached as high as 21.7%, much higher than the 14.1 and 8.89% in the Renhuai and Duyun production regions, respectively. *Schizosaccharomyces* may play a crucial role in carbohydrate metabolism and flavor compound synthesis during the fermentation of Jiang-flavored baijiu. Specifically, the literature indicates that *Schizosaccharomyces* has a special acetic acid-tolerant ability to produce Jiang-flavored baijiu. Moreover, it degrades high concentrations of acetic acid by enhancing the mevalonate pathway. Simultaneously, it increases the levels of 227 flavor-related metabolites to improve the taste and quality of baijiu (Song et al., 2019; Du et al., 2021). The high abundance of *Schizosaccharomyces* in the Bijie production region may be associated with the “invasion” of microorganisms from the strong-flavor baijiu workshop. Certain fungi or their metabolites in strong-flavor baijiu brewing may provide favorable conditions for the growth and reproduction of *Schizosaccharomyces* or inhibit the growth of other competing fungi, thus enabling *Schizosaccharomyces* to dominate in the fermented grains of the Bijie production region. The dominance of fungi unique to the Bijie production region, such as *Paecilomyces* (13.4%) and *Pichia* (8.03%), further demonstrates the distinctiveness of the fungal community structure in this region. This is likely closely related to the combined effect of the local environment and microorganisms from the strong-flavored baijiu workshop.

### 3.2 α-diversity analysis

A comparison of bacterial α-diversity among the three origins revealed that the indices characterizing species richness and evenness were higher in the Bijie region than in the other two regions. Notably, the bacterial communities in the Bijie samples exhibited more pronounced richness and evenness. Conversely, the bacterial diversity of the bacteria in the Renhuai region showed a more balanced pattern than that of the other two regions.

The diversity indices for fungi resembled those for bacteria. The comparison indicated that the Shannon, Simpson, and Pielou evenness indices in the Bijie production region were higher than those of the other regions, with the difference from Duyun reaching statistical significance. Moreover, the Chao1 index of the Renhuai-producing area was higher than that of the other two areas, whereas the Shannon and Pielou evenness indices of the Duyun-producing area were lower (see Table 1).

**Table 1:** α-diversity indices of bacteria and fungi in the baijiu samples investigated in this study.

Microbial taxa	Samples	Chao1	Shannon	Simpson	Pielou evenness
Bacteria	A	2205.67 ± 52.25b	4.72 ± 0.02a	0.96 ± 0.01b	0.61 ± 0.01a
	B	2633.53 ± 114.28a	4.92 ± 0.09a	0.97 ± 0.01a	0.62 ± 0.01a
	C	1644.69 ± 212.53c	4.05 ± 0.17b	0.92 ± 0.01b	0.55 ± 0.01a
Fungi	A	709.40 ± 24.20a	3.35 ± 0.33a	0.87 ± 0.03a	0.51 ± 0.05a
	B	667.18 ± 6.97a	3.82 ± 0.06a	0.93 ± 0.01a	0.59 ± 0.01a
	C	498.79 ± 16.6b	2.01 ± 2.01b	0.62 ± 0.21a	0.32 ± 0.11b

In the samples column, values with the same letter indicate no significant difference, while different letters indicate significant differences.

The substantial differences in microbial diversity among the different production areas imply that, during the second-round fermentation, geographical location disparities influence the structure and composition of microorganisms, despite the differences in the brewing process not yet being fully manifested. Variation in microbial diversity across different production areas may be attributed to the synergistic effects of multiple factors. For instance, the microbial diversity in the Bijie production region was notably higher than that in the other regions. Besides its distinct geographical environment, its close proximity to the workshops of strong-flavored baijiu is a crucial factor that cannot be overlooked. During the brewing of strong-flavor baijiu, unique microbial flora are continuously released into the environment. These microorganisms may have infiltrated the Jiang-flavored baijiu-brewing environment of the Bijie region via air circulation, equipment transfer, and personnel movement. The more balanced diversity in the Renhuai region may be ascribed to local microorganisms undergoing co-adaptation and co-evolution during the brewing process, thereby establishing a stable and harmonious microbial ecosystem. Although the microbial diversity in Renhuai was not the highest, the microbial community structure optimized over time. Metabolic cooperation between microorganisms achieves a delicate equilibrium, enabling them to stably and efficiently mediate various biochemical reactions during the Jiang-flavor baijiu-brewing process. This ensures the stability and uniqueness of Jiang-flavored baijiu.

### 3.3 Species variation and beta diversity

**Figure 2:**
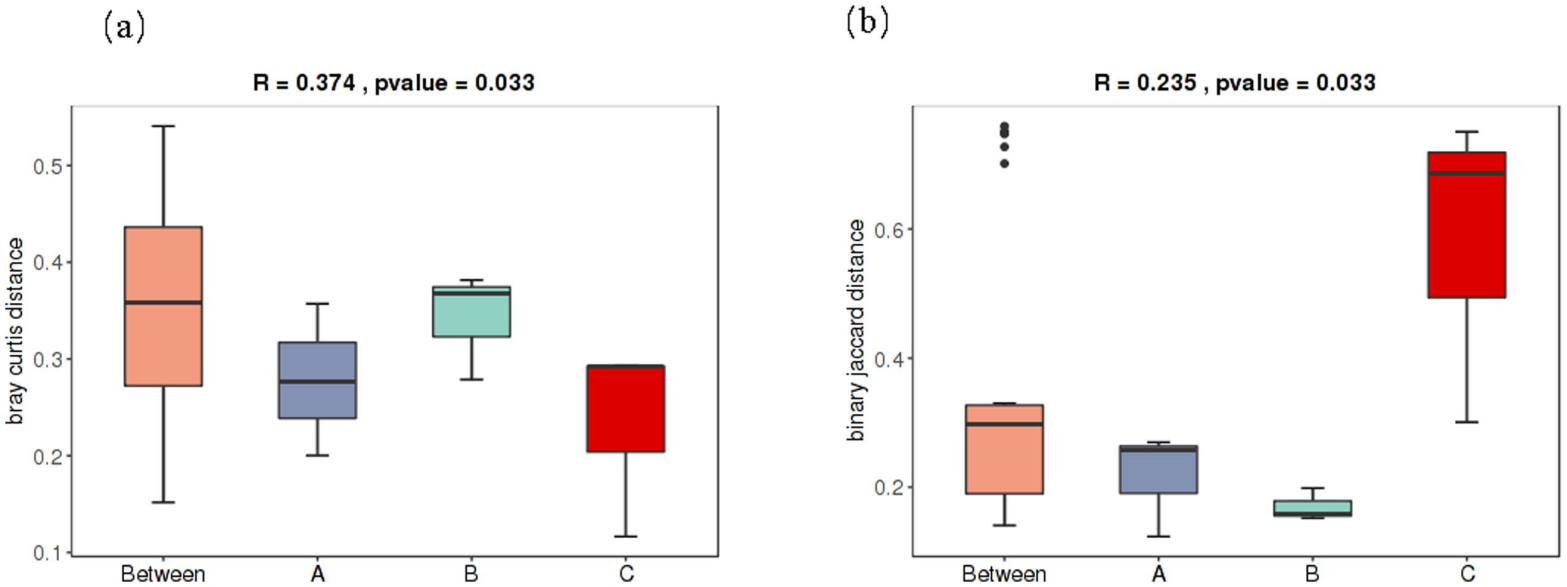
Box plot of the binary Jaccard distance between **(a)** bacterial and **(b)** fungal samples at the genus level.

ANOSIM represents a statistical approach applied to analyze similarities among groups of multidimensional data. This approach was employed to determine the presence of significant differences between bacterial and fungal communities in baijiu sourced from distinct production areas. As depicted in Figures 2a,b, the analysis results demonstrate that the *p*-values for both bacterial and fungal communities are less than 0.05. This indicates the presence of statistically significant differences in the grouping of bacterial and fungal communities in baijiu from different production areas. In other words, the microbial communities in baijiu from different production areas did not exhibit random distribution; instead, they were influenced by specific factors and displayed evident grouping characteristics. Simultaneously, the R-values ranged from > 0.25 to < 0.5. This finding implies a certain degree of variation among the three production regions; however, the difference was relatively minor. This could potentially be ascribed to the similar microbial compositions of the same soy-sauce-flavored baijiu resulting from comparable production processes. Further comparisons revealed that the R-value of the bacterial community was higher than that of the fungal community. This implies that differences in the structure of the bacterial community were more pronounced than those of the fungal community in spirits from different production regions. This phenomenon may be attributed to the higher sensitivity of bacteria to environmental factors. Subtle alterations in the geographical environment and brewing processes across different regions can readily induce changes in bacterial community structure. In contrast, fungi may possess greater environmental adaptability, resulting in a more stable community structure.

Although previous relevant studies have pointed out that bacteria primarily originate from Daqu, and fungi originate from the brewing environment (Zhang, 2021), this perspective requires further validation and refinement within the context of diverse production areas. The microbial backgrounds in the Daqu production process, raw materials, and brewing environments differ across various production regions. Such differences can influence the proportion of bacterial and fungal sources as well as the actual community composition. Uncovering these disparities will allow for a more precise understanding of the characteristics of microbial communities in distinct production regions. Furthermore, this study provides a broader perspective for subsequent research on the relationship between microbial communities and baijiu quality.

**Figure 3:**
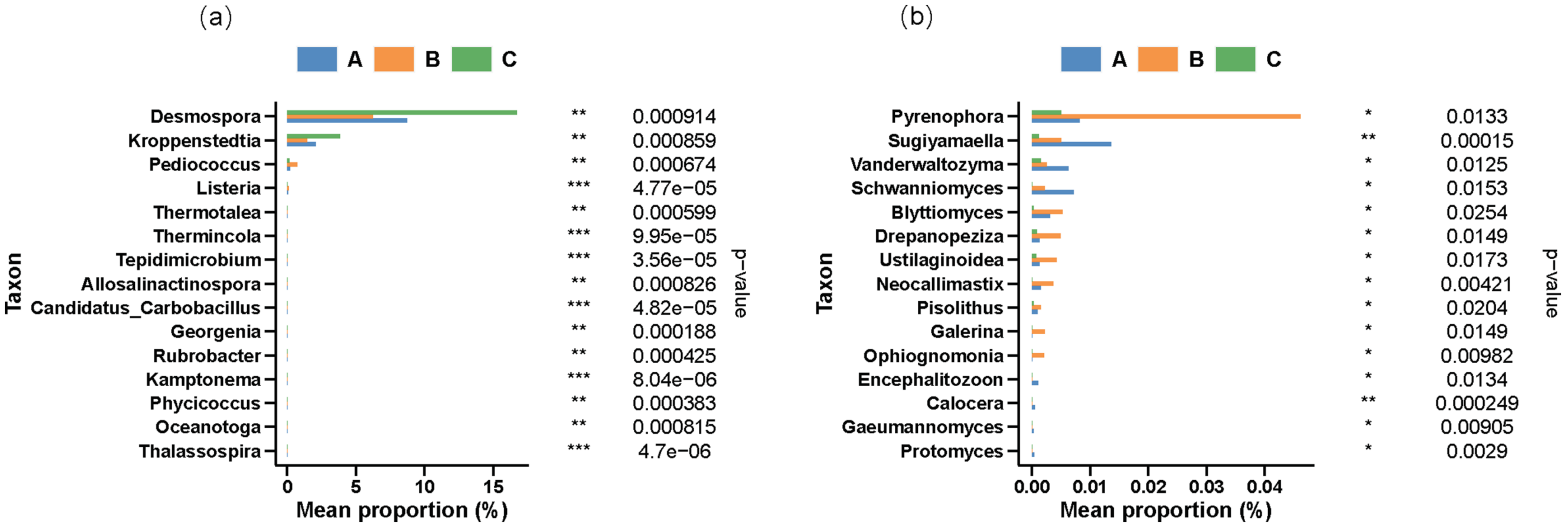
Rank-sum test differential species abundance histogram of **(a)** bacteria and **(b)** fungi at the genus level.

To further delineate the differences among genera, the rank-sum test was used to compare the abundances of various species. As shown in Figure 3a, the 15 most significant bacterial genera were *Desmospora*, *Kroppenstedtia*, *Pediococcus*, *Listeria*, *Thermotalea*, *Thermincola*, *Tepidimicrobium*, *Allosalinactinospora*, *Candidatus*, *Carbobacillus*, *Georgenia*, *Rubrobacter*, *Kamptonema*, *Phycicoccus*, *Oceanotoga*, and *Thalassospira*. The abundance ratios of *Desmospora* and *Kroppenstedtia* were significantly higher in the Duyun production region than in the other two regions. The abundance of *Pediococcus* and *Listeria* in the Bijie production area was notably higher than in the Renhuai production area. *Desmospora*, a prevalent microorganism in high-temperature Daqu in the Sichuan and Guizhou provinces, may be involved in specific substance transformation processes. Its metabolites may play a pivotal role in shaping the flavor of Duyun Jiang-flavored baijiu. For example, they may contribute to the production of certain esters or alcohols, endowing baijiu with a distinctive aroma and taste.

The high abundance of *Kroppenstedtia* may have influenced the energy metabolism pathway in fermented grains. It has been proposed that this strain is the primary factor for the differences in aldehyde and ketone contents observed. It is primarily involved in lipid synthesis and enzymatic degradation. Consequently, during fermentation, the energy supply and utilization patterns of the fermented grains in this region diverge from those in other regions, thereby affecting the overall metabolic activities of the microbial communities (Yan et al., 2025). *Pediococcus* is a common bacterium found in high-temperature Daqu. Its substantial presence in Bijie is likely associated with the lactic acid content of the fermented grains. This change in acidity not only impacts the growth and metabolism of other microorganisms but may also participate in the formation of flavor substances in Jiang-flavored baijiu. For instance, it can undergo esterification with alcohols to generate esters with unique flavors (Li et al., 2024).

Among the fungi, the 15 species that reached a significant level in the Bijie production area are *Pyrenophora*, *Sugiyamaella*, *Vanderwaltozyma*, *Schwanniomyces*, *Blyttiomyces*, *Drepanopeziza*, *Ustilaginoidea*, *Neocallimastix*, *Pisolithus*, *Galerina*, *Ophiognomonia*, *Encephalitozoon*, *Calocera*, *Gaeumannomyces*, and *Protomyces*. The abundances of *Pyrenophora* and *Blyttiomyces* in the Bijie region were higher than those in the other two regions, whereas the abundances of *Sugiyamaella* and *Vanderwaltozyma* in Renhuai were higher than those in the other two areas. Previous studies have indicated that *Sugiyamaella* can be detected in the cellar residue of Jiang-flavored baijiu (Wei et al., 2023). These differences in fungal composition may play crucial roles in the synthesis of flavor substances. Their unique metabolic pathways may yield compounds with special aromas, imparting signature flavor characteristics to the Jiang-flavored baijiu in each region.

### 3.4 Analysis of the potential functions of microorganisms

**Figure 4:**
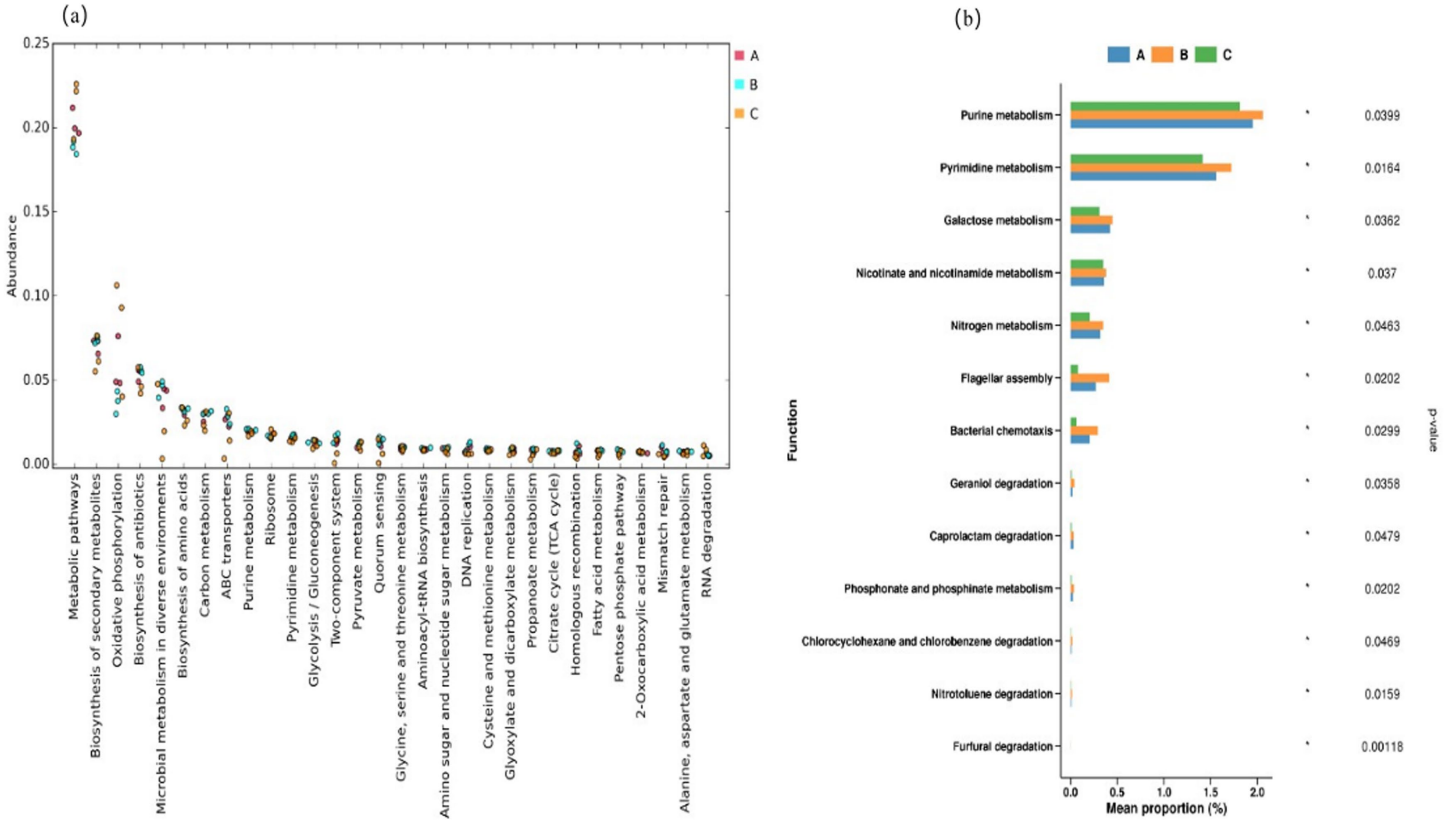
Scatter plot of **(a)** the classification of KEGG metabolic pathways at the tertiary level and **(b)** bar chart of the differentially functional genes.

Based on the KEGG database, the metagenomic data of microbial communities in the three types of fermented grains were analyzed according to their modular functions. As depicted in Figure 4a, at KEGG level III, metabolism-related genes exhibited the highest abundance and were predominant. In terms of the overall expression level, the ranking was Duyun > Renhuai > Bijie.

Microbial metabolic reactions lie at the core of the brewing process of Jiang-flavored baijiu. The high expression of metabolism-related genes implies that microorganisms in the Duyun region may possess stronger basic metabolic capacity and can more efficiently utilize raw materials for substance conversion than those in the other regions (Kanehisa et al., 2017, 2022). This phenomenon may be associated with the relatively unique microbial community structure of the Duyun production area. Conversely, the abundance of metabolism-related genes in the Bijie production area was relatively low. Despite its high microbial diversity, the complexity of the microbial community structure may lead to resource competition or functional redundancy during the metabolic processes of some microorganisms, thereby affecting overall metabolic efficiency. Collectively, these data suggest that differences in the species and abundance of the dominant microorganisms may determine the overall expression levels of metabolic genes.

The biosynthesis of secondary metabolites ranked second, with only minor differences among the three production sites. The functional expression of genes related to oxidative phosphorylation was similar to that of metabolic pathways, with the expression levels following the order Duyun > Renhuai > Bijie. The minimal differences observed in the biosynthesis of secondary metabolites among the three production areas suggest that microorganisms in different areas have comparable abilities to synthesize secondary metabolites, such as flavor substances, during the brewing process. This could be attributed to the common requirements of the Jiang-flavored baijiu-brewing process, which enables microorganisms in different production areas to maintain relative stability in this crucial function.

The expression levels of genes associated with microbial metabolism in diverse environments were similar in the Renhuai and Bijie production areas and higher than those in the Duyun production area. This indicates that the microorganisms in Renhuai and Bijie have stronger adaptability and metabolic regulation capabilities in response to environmental changes. In the Bijie production area, the special environment close to a distillery may prompt microorganisms to develop more flexible metabolic strategies to adapt to complex and variable brewing environments than those in the other areas. Specifically, the abundance of metabolism-related genes was significantly higher in the Duyun production area than in the Renhuai and Bijie production areas.

Through comparative analysis of differentially functional genes, purine metabolism, pyrimidine metabolism, galactose metabolism, nicotinate and nicotinamide metabolism. The expression of genes related to metabolism, nitrogen metabolism, flagellar assembly, and bacterial chemotaxis followed the order Bijie > Renhuai > Duyun, with the difference among the three production areas reaching statistical significance. This may be related to the unique microbial community structure in the Bijie production area and the influence of microorganisms in fragrance workshops. The introduction of microorganisms into the strong-flavor baijiu workshop may activate new metabolic pathways and gene resources in the Bijie production region and enrich the metabolic function of microorganisms. Simultaneously, the Bijie production area has high microbial diversity, and the cooperative relationship between different microorganisms may also be conducive to the optimization of these metabolic pathways.

### 3.5 Multivariate statistical analysis of functional genes and environmental factors

**Figure 5:**
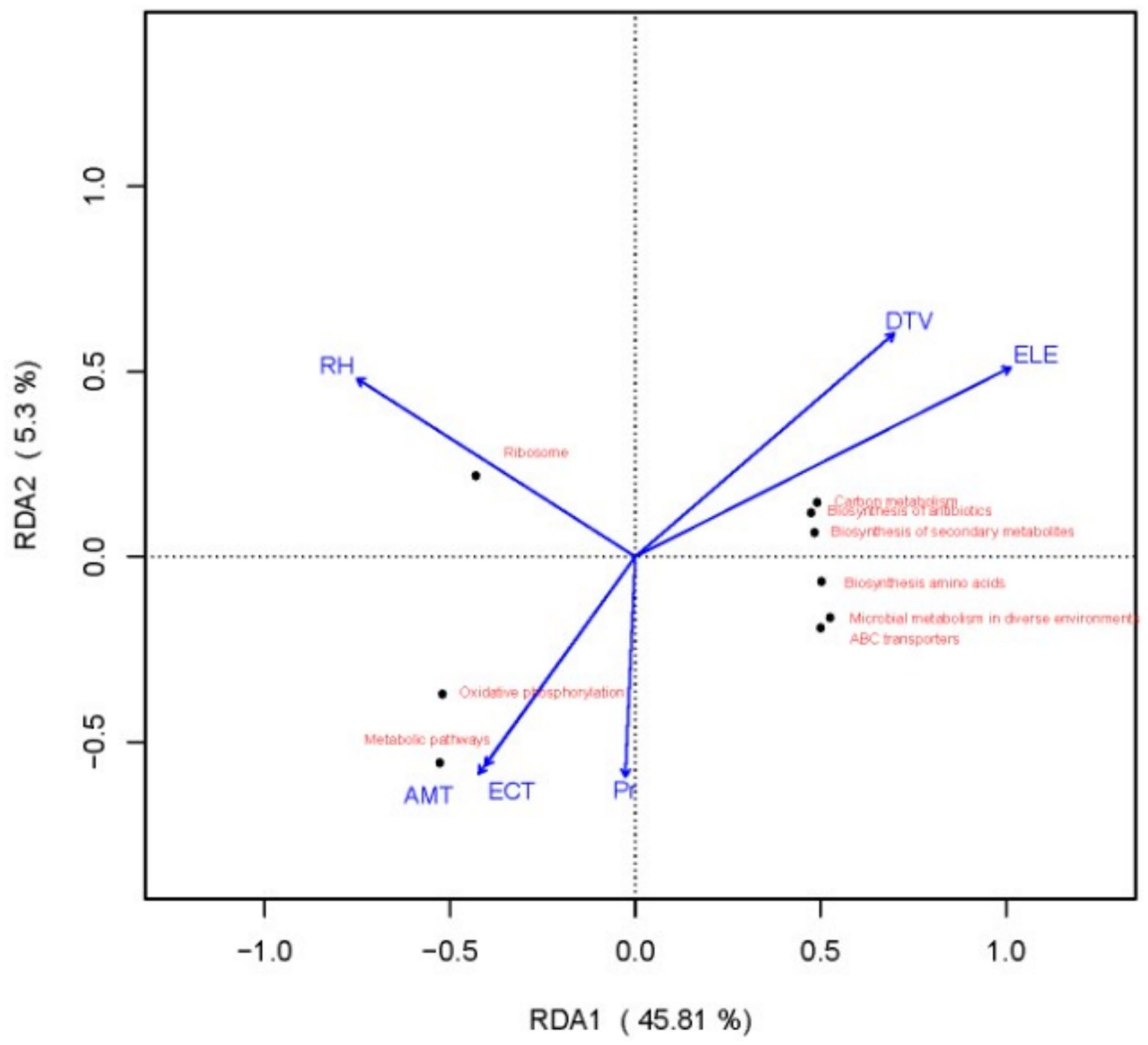
Redundancy analysis of the relationships between Kyoto Encyclopedia of Genes and Genomes functional genes and environmental factors.

The redundancy analysis (RDA) results, as shown in Figure 5, indicate that the first and second axes accounted for 45.81 and 5.3% of the variation in the response variables, respectively, with a cumulative explanatory power of 51.11%. These findings suggest that environmental factors, including relative humidity (RH), diurnal temperature variation (DTV), elevation (ELE), mean annual temperature (AMT), extremely low temperature (ECT), and annual precipitation (Pr), significantly affected the functional gene profiles of microbial communities in fermented grains.

Specifically, all functional genes involved in carbon metabolism, antibiotic biosynthesis, and secondary metabolite biosynthesis were positively correlated with the ELE. This finding suggests that the expression of these three functional gene sets increases along the elevational gradient. Microbial community composition not only influences disparities in functional genes but also affects the preference of microorganisms for carbon and nitrogen sources (Jiang et al., 2021). This phenomenon may be attributed to the fact that elevation is associated with changes in temperature and oxygen levels. In high-altitude regions, relatively low temperatures and oxygen contents prompt microorganisms to develop more efficient carbon metabolism strategies to store adequate energy and materials. Simultaneously, to compete for survival in relatively harsh environments, microorganisms may enhance the synthesis of antibiotics and secondary metabolites to resist invasion by other microorganisms.

There was a significant positive correlation between ribosome-related functional genes and RH. That is, as the RH increases, the expression of ribosome functional genes also increases, which may be because water is crucial for the physiological activity of microbial cells. A higher RH helps maintain water balance within microbial cells, providing a suitable environment for the normal assembly of ribosomes and protein synthesis.

Oxidative phosphorylation and metabolic pathways were significantly positively correlated with the AMT and ECT, but negatively correlated with the DTV and ELE. Appropriate AMT and ECT conditions offer a suitable temperature range for the activity of microbial metabolic enzymes, thereby promoting oxidative phosphorylation and efficient operation of overall metabolism. Conversely, low-temperature and low-oxygen environments resulting from a large daily temperature difference and increasing altitude undermine the stability of microbial metabolic pathways.

### 3.6 Characteristics of microbial carbon metabolism

**Figure 6:**
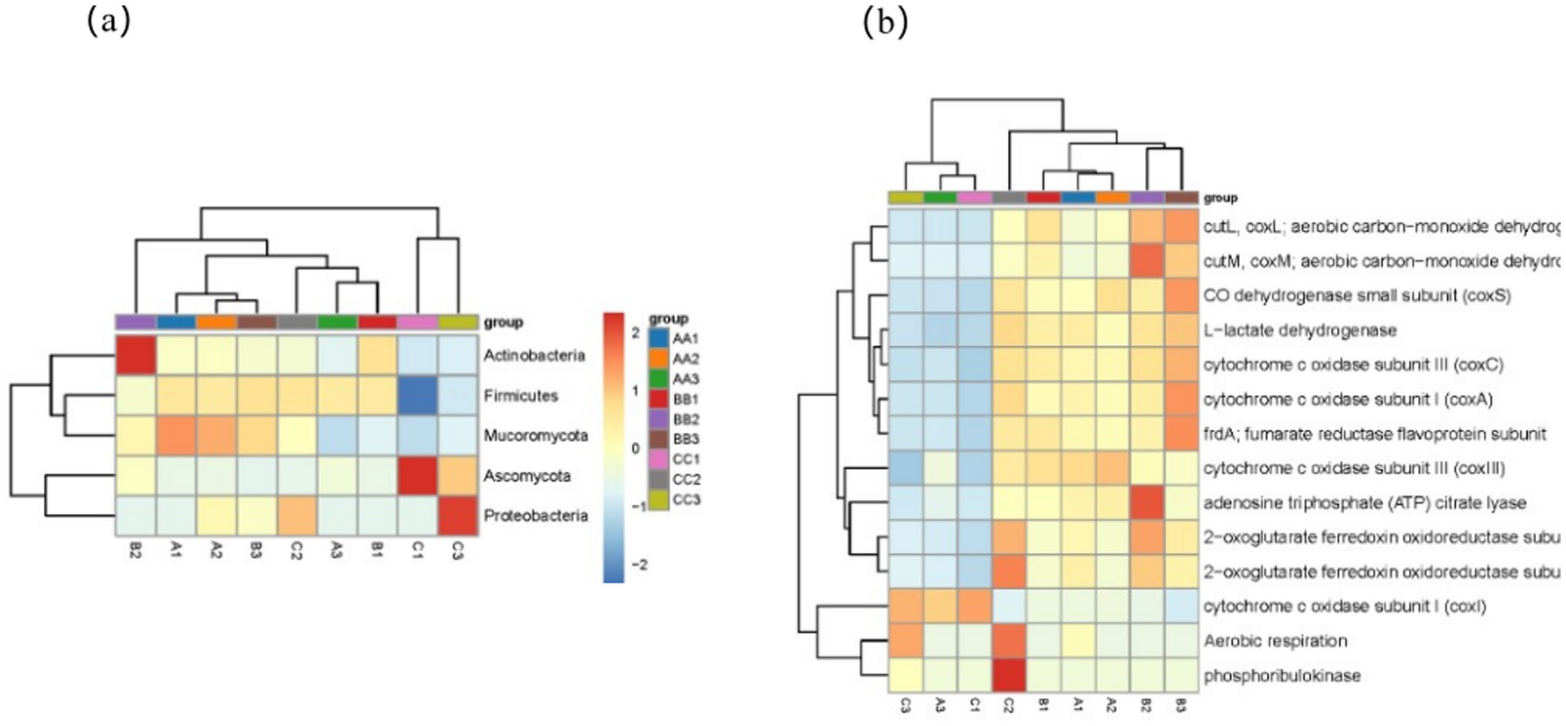
The main microbial phyla and the top 20 types of carbon metabolism in terms of gene abundance. **(A)** Taxonomic distribution of main microbial phyla. **(b)** Functional classification of top carbon metabolism types.

At the phylum level (Figure 6a), the microorganisms associated with carbon metabolism in the samples of second-round fermented grains from the three production areas were analyzed. The results indicated that Actinobacteria were the dominant phylum, followed by Proteobacteria and Chloroflexi. Additionally, Acidobacteria and Archaea showed a relatively high abundance. These dominant phyla likely play crucial roles in carbon metabolism in fermented grains.

Statistical analysis was then conducted on the functional genes involved in the carbon metabolism pathway (Figure 6b) to identify the genes with high abundance during carbon metabolism. The results revealed that during the carbon cycle of the two rounds of fermentation of grains from the three production areas, genes with high abundance primarily included aerobic carbon monoxide dehydrogenase small subunit (*coxs*), lactate dehydrogenase, cytochrome C oxidase subunit (*coxC*), cytochrome C oxidase subunit 1 (*coxA*), and fumarate reductase flavoprotein subunit. Following these were ATP citrate lyase, 2-oxoglutarate ferredoxin oxidoreductase subunit, and phosphoribulokinase. These genes with high abundance play a pivotal role in carbon metabolism. Genes encoding aerobic carbon monoxide dehydrogenase may be involved in utilizing specific carbon sources. The CO dehydrogenase small subunit is closely associated with energy metabolism and electron transfer, enabling microorganisms to acquire energy and perform material transformations within the grain environment. The similarities and differences in the abundance of these genes among fermented grains from the different production areas reflect the adaptive evolution of microbial communities to the environments of various production regions. Further research should be carried out to explore the correlations between these genes, the fermentation characteristics of fermented grains, and baijiu quality, thus providing a theoretical foundation for optimizing brewing technology.

Actinobacteria, the dominant phylum in carbon metabolism, likely play a pivotal role in the carbon cycle of fermented grains. Actinobacteria exhibit rich metabolic diversity and can utilize a wide range of complex carbon sources (Wang L. et al., 2021). This metabolic versatility may account for the dominance of carbon metabolism. Actinobacteria may also engage in crucial reactions, such as aerobic carbon monoxide dehydrogenase-mediated processes and lactate dehydrogenation by producing various enzyme systems. This is consistent with genes encoding aerobic carbon monoxide dehydrogenase and lactate dehydrogenase, which are highly abundant during carbon metabolism, suggesting a close link between the microbial community structure and functional genes. Proteobacteria and Chloroflexi may participate in the different phases of carbon metabolism. Their synergistic actions probably contribute to maintaining a stable carbon cycle within fermented grains.

When comparing the three production areas, despite the similarities in phylum-level composition and functional gene types related to carbon metabolism, the relative abundance of microbial communities and subtle differences in gene expression in each area may result in variations in carbon metabolic efficiency and products. These disparities are likely due to the influence of environmental factors on microbial functional genes in different production areas, which can indirectly affect carbon metabolism. The distinct climate and geographic factors in the three production areas may have caused the microbial community to adapt its carbon metabolism pathways, affecting substance transformation during the brewing process, and ultimately manifest in the flavor and quality of the Jiang-flavored baijiu.

Nevertheless, knowledge regarding the specific mechanisms of action of these different bacteria in the production of Jiang-flavored baijiu remains limited. In subsequent studies, key differential bacteria should be isolated using pure culture techniques, and metabolomic technology should be employed to analyze the changes in the metabolites of these bacteria during the fermentation of grains. This would enable the identification of the specific contributions of these key differential bacteria to the formation of flavoring substances in Jiang-flavored baijiu.

## 4 Policy recommendations

First, governments and associations should guide regional baijiu producers to enhance the differentiated competitiveness of their products. Jiang-flavored baijiu brewed in different locations exhibits distinct flavors; before the rise of Jiang-flavored baijiu, various regions had unique local baijiu flavor types, such as the “Yun” flavor from Duyun Distillery and the “Dong” flavor from Dongjiu. Governments and associations should encourage producers to continue manufacturing and promote their characteristic baijiu products, fostering differentiated competition to replace the current homogeneous competition model.

Second, when implementing environmental protection initiatives, local microbial communities should be prioritized for conservation. It is recommended that the government collaborate with associations to establish a Baijiu Microbial Germplasm Resource Bank that systematically documents microbial genetic data in baijiu with characteristic regional flavors. Using metagenomic technology, core functional microbial communities should be analyzed to clarify the correlation between their metabolites and the presence of flavor compounds, providing a scientific basis for the standardized production of local baijiu flavor types.

Third, the government, associations, and baijiu producers should explore the protection and inheritance of traditional brewing techniques while enhancing the joint promotion of raw materials, environments, aromas, and processes of different origins. Using locally grown raw materials and highlighting differences in water sources, environments, and cultural backgrounds can emphasize product uniqueness. Universities and research institutions should also continuously study the factors influencing the relationship between the production origins and flavors of baijiu.

Fourth, associations should strengthen their research efforts to conduct flavor evaluations and determine the demand for Jiang-flavored baijiu from diverse consumer groups across the nation. This will guide the development of differentiated Jiang-flavored baijiu enterprises in different regions, avoiding issues such as microbial community environmental damage, homogeneous vicious competition, and misaligned consumer market targeting caused by blind trend-following, fostering the generation of regional characteristic brands.

Fifth, in addition to large-scale distilleries, many regions have locally renowned “artisanal” baijiu produced in villages and towns, which are crafted in unique local environments. Numerous small-scale baijiu-processing facilities have been established through the long-term optimization of brewing techniques and standardizing baijiu production. Although these products are less famous than Jiang-flavored baijiu, they offer specific advantages, such as unique flavors, acceptable quality, and affordable prices, forming an operational model with low investment, low risk, and quick returns. Promoting this model at appropriate scales can increase the planting income of farmers and enhance the ability of distillery operators to withstand market risks.

## 5 Conclusion

This study focused on the microbial communities in the second-round fermented grains of Jiang-flavored baijiu. Using multiple analytical and statistical methods, we explored the bacterial and fungal community structures, their functional genes, and their correlations with environmental factors. In terms of microbial community composition, many bacterial and fungal genera were identified in the three production regions. The dominant species were similar across regions, but their relative abundances varied significantly. *α*-diversity analysis showed that Bijie had higher microbial diversity in several indices, highlighting the impacts of geographical location and the presence of nearby strong-flavor baijiu workshops. ANOSIM analysis and the rank-sum test revealed significant differences in microbial community groupings among production areas and identified genera with large differences in abundance. A study of potential microbial functions indicated that Duyun had the highest abundance of metabolism-related genes at the KEGG tertiary level. Redundancy analysis revealed six environmental factors, including RH and ELE, exerting complex influences on microbial genes. A carbon metabolism study found that Actinobacteria and Ascomycetes are crucial for carbon metabolic processes. Based on the above results, we propose that governments and associations guide local baijiu enterprises in developing products according to their regional environments. In particular, they should promote flavor profiles that already have a local reputation, thereby enabling baijiu products to develop unique characteristics. This approach can foster differentiated and healthy competition by replacing ineffective homogeneous competition.

## Data Availability

The datasets presented in this study can be found in online repositories. The names of the repository/repositories and accession number(s) can be found at: data are available from https://www.ncbi.nlm.nih.gov/ under accession PRJNA1286790.
