# Supplementary material for: Metagenomics-based analysis of microbial community structure and functional differences in fermented grains of Jiang-flavored baijiu from different production regions and policy recommendations for industrial development
Source: Front Microbiol. 2025 Jul 16;16:1619035. doi: 10.3389/fmicb.2025.1619035 (PMC12307490; doi:10.3389/fmicb.2025.1619035)
Supplement: Supplementary file 1 [file Table_1.docx]

| **Sample ID** | **Clean data base(bp)** | **Number of Reads** | **GC(%)** | **Q20(%)** | **Q30(%)** |
| --- | --- | --- | --- | --- | --- |
| **A1** | 6,104,727,874 | 40,143,750 | 43.24 | 98.1 | 94.44 |
| **A2** | 6,155,820,642 | 40,653,138 | 42.99 | 98.14 | 94.52 |
| **A3** | 6,411,315,372 | 42,315,898 | 37.51 | 98.02 | 94.13 |
| **B1** | 6,027,750,772 | 39,630,836 | 40.68 | 97.93 | 93.91 |
| **B2** | 6,087,843,002 | 39,980,032 | 45.76 | 97.71 | 93.77 |
| **B3** | 6,266,589,580 | 41,361,040 | 41.04 | 98.03 | 94.26 |
| **C1** | 6,318,987,148 | 41,806,034 | 37.31 | 98.13 | 94.32 |
| **C2** | 6,258,271,804 | 41,176,606 | 44.47 | 98.06 | 94.42 |
| **C3** | 6,131,333,730 | 40,546,058 | 38.48 | 98.11 | 94.3 |

S1 Statistical Evaluation of Sample Sequencing Data
